# When Stroke Is Not a Stroke: Wernicke’s Encephalopathy in a Patient With Chronic Alcohol Misuse

**DOI:** 10.7759/cureus.102099

**Published:** 2026-01-22

**Authors:** Junaid Khan, Anusha Jahid, Sadia Ali, Carmen Constantin

**Affiliations:** 1 Stroke Medicine, United Lincolnshire Teaching Hospitals NHS Trust, Lincoln, GBR; 2 Internal Medicine, University Hospitals of Derby and Burton NHS Foundation Trust, Derby, GBR

**Keywords:** acute neurological deterioration, alcohol misuse, stroke mimic, thiamine deficiency, wernicke–korsakoff syndrome, wernicke’s encephalopathy

## Abstract

Wernicke’s encephalopathy (WE) is a neurological emergency caused by thiamine deficiency and is a recognised stroke mimic. While prior reports often describe subacute presentations, we present a uniquely instructive case characterised by abrupt neurological collapse, with a rapid decline in Glasgow Coma Scale from 14 to 3 within 48 hours, closely simulating catastrophic cerebrovascular disease. Initial computed tomography was unremarkable; however, magnetic resonance imaging demonstrated classic, symmetrical involvement of the medial thalami, periaqueductal grey matter, tectal plate, mammillary bodies, and pyramidal tracts, consistent with acute WE. High-dose intravenous thiamine was initiated promptly, resulting in marked radiological improvement within five days. Despite recovery of consciousness and imaging resolution, the patient exhibited persistent and severe cognitive impairment on follow-up (Montreal Cognitive Assessment score 12/30), including prominent memory deficits. This case highlights an important and under-emphasised clinical lesson: radiological reversibility in WE does not reliably predict cognitive recovery, particularly following extreme presentations with abrupt coma. The report reinforces the need for immediate empirical thiamine administration in patients with acute unexplained neurological deterioration and risk factors for thiamine deficiency and cautions against interpreting early imaging improvement as evidence of a favourable neurological outcome.

## Introduction

Wernicke’s encephalopathy (WE) is a life-threatening neuropsychiatric syndrome resulting from thiamine (vitamin B1) deficiency and constitutes a true medical emergency [1]. Although traditionally described by a triad of encephalopathy, gait ataxia, and ophthalmoplegia, fewer than one-third of patients present with all three features [2], contributing to frequent under-recognition and delayed treatment [3]. Autopsy studies suggest that up to 80% of cases may be missed during life [4].

Chronic alcohol misuse is the most common predisposing factor due to reduced dietary intake, impaired intestinal absorption, and altered hepatic metabolism of thiamine [5]. Additional risk factors include malnutrition, prolonged immobility, liver disease, and acute medical illness [6]. Importantly, WE can present with acute neurological decline mimicking stroke, particularly when there is sudden deterioration in consciousness [7].

Magnetic resonance imaging (MRI) plays a key role in diagnosis, typically demonstrating symmetrical lesions involving the medial thalami, periaqueductal grey matter, mammillary bodies, and tectal plate [8]. Early high-dose parenteral thiamine therapy can reverse both clinical and radiological abnormalities, whereas delayed treatment may result in irreversible neurological injury [9]. However, the relationship between radiological recovery and long-term neurological outcome remains incompletely understood.

This abstract was presented as a poster at the World Congress of Neurology 2025 in Seoul, South Korea, from October 12 to 15, 2025.

## Case presentation

A 63-year-old man was brought to the emergency department after being found confused and immobile at home. He lived alone and had a long-standing history of heavy alcohol use. His medical history was significant for bilateral above-knee amputations secondary to peripheral arterial disease. Collateral history revealed poor nutritional intake, social isolation, and progressive behavioural and cognitive changes over several weeks prior to admission.

On initial assessment, the patient was confused and disoriented with a Glasgow Coma Scale (GCS) score of 14/15 (E4, V4, M6). Neurological examination demonstrated dysarthria, truncal ataxia, and impaired attention and memory. There was no ophthalmoplegia or nystagmus. Cranial nerve examination was otherwise unremarkable, and no focal limb weakness was identified. Vital signs were stable. Bilateral conjunctival injection with purulent discharge was noted.

Laboratory investigations revealed significantly deranged liver function tests (alanine aminotransferase 186 U/L, aspartate aminotransferase 212 U/L, alkaline phosphatase 268 U/L, gamma-glutamyltransferase 647 U/L, and bilirubin 58 µmol/L), mild thrombocytopenia, elevated inflammatory markers, and hypomagnesemia. A non-contrast CT scan of the brain revealed no acute abnormalities. Due to concerns for infection, intravenous fluids and empirical antibiotics were commenced.

Given the acute encephalopathy, an initial differential diagnosis included infection or sepsis (including encephalitis), toxic metabolic encephalopathy, hepatic encephalopathy, non-convulsive status epilepticus, and acute cerebrovascular disease. Empirical intravenous fluids and antibiotics were commenced while investigations were ongoing.

Within 48 hours of admission, the patient experienced a sudden neurological deterioration, with a rapid decline in GCS from 14 to 3 (E1, V1, M1). He exhibited spontaneous limb movements but no response to painful stimuli. Brainstem reflexes, including pupillary and corneal reflexes, were preserved. No clinical seizure activity was observed. Non-convulsive status epilepticus was considered unlikely in the absence of seizure activity or fluctuating consciousness, and preserved brainstem reflexes argued against primary brainstem structural pathology.

Infectious causes were reconsidered; however, the patient remained haemodynamically stable, and inflammatory markers did not significantly worsen, with no infectious source identified. There was no clinical or biochemical evidence of hypoglycaemia, electrolyte disturbance, or drug intoxication. Given the sudden loss of consciousness, an acute cerebrovascular event was initially considered.

**Figure 1:**
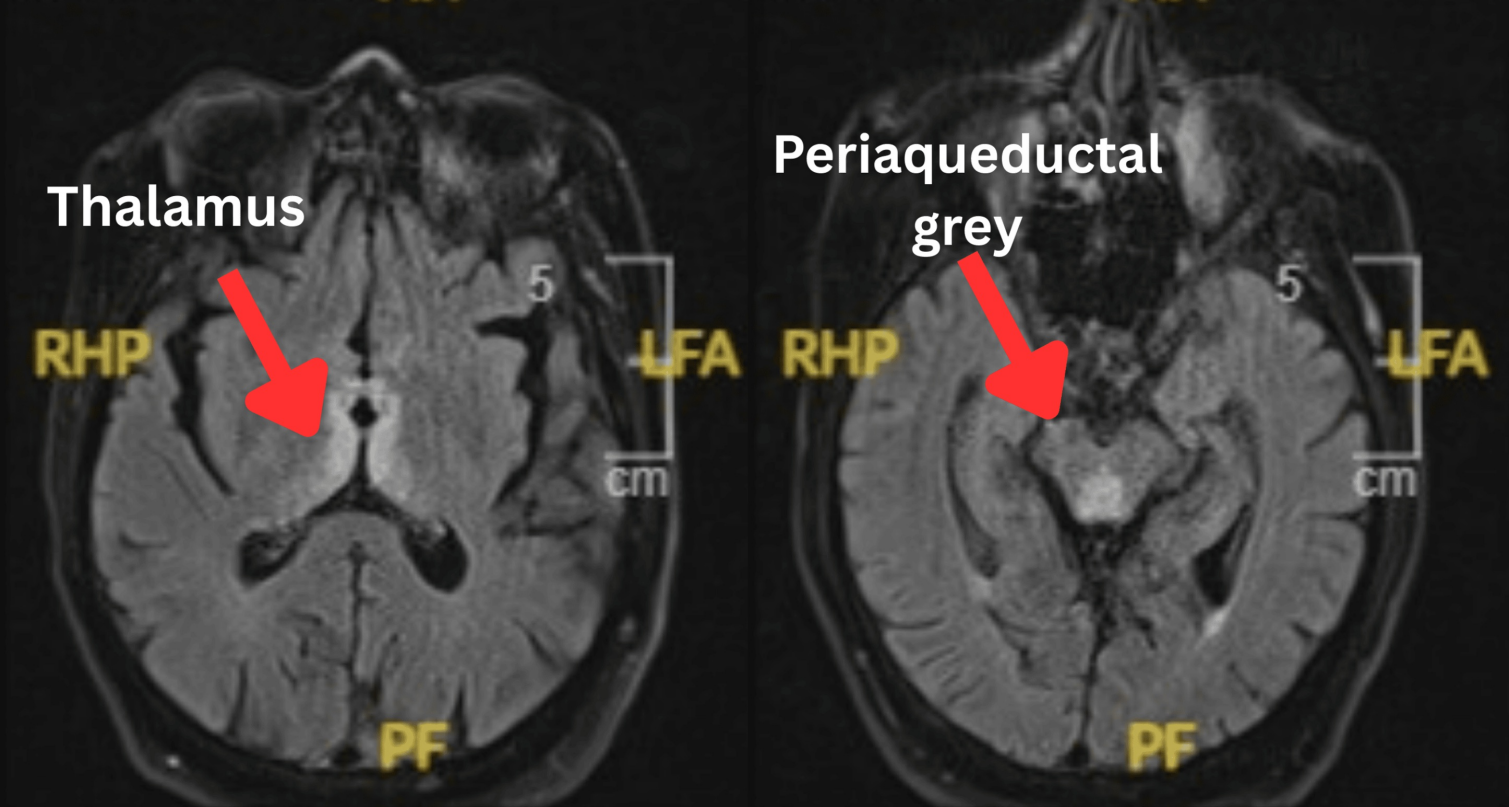
MRI brain (axial FLAIR) demonstrating symmetrical hyperintense signal involving the medial dorsal thalami, periaqueductal grey matter, tectal plate, and mammillary bodies, characteristics of acute WE. MRI: magnetic resonance imaging; FLAIR: fluid-attenuated inversion recovery; LFA: left frontal area; RHP: right hemispheric parietal; PF: posterior fossa; WE: Wernicke’s encephalopathy.

**Figure 2:**
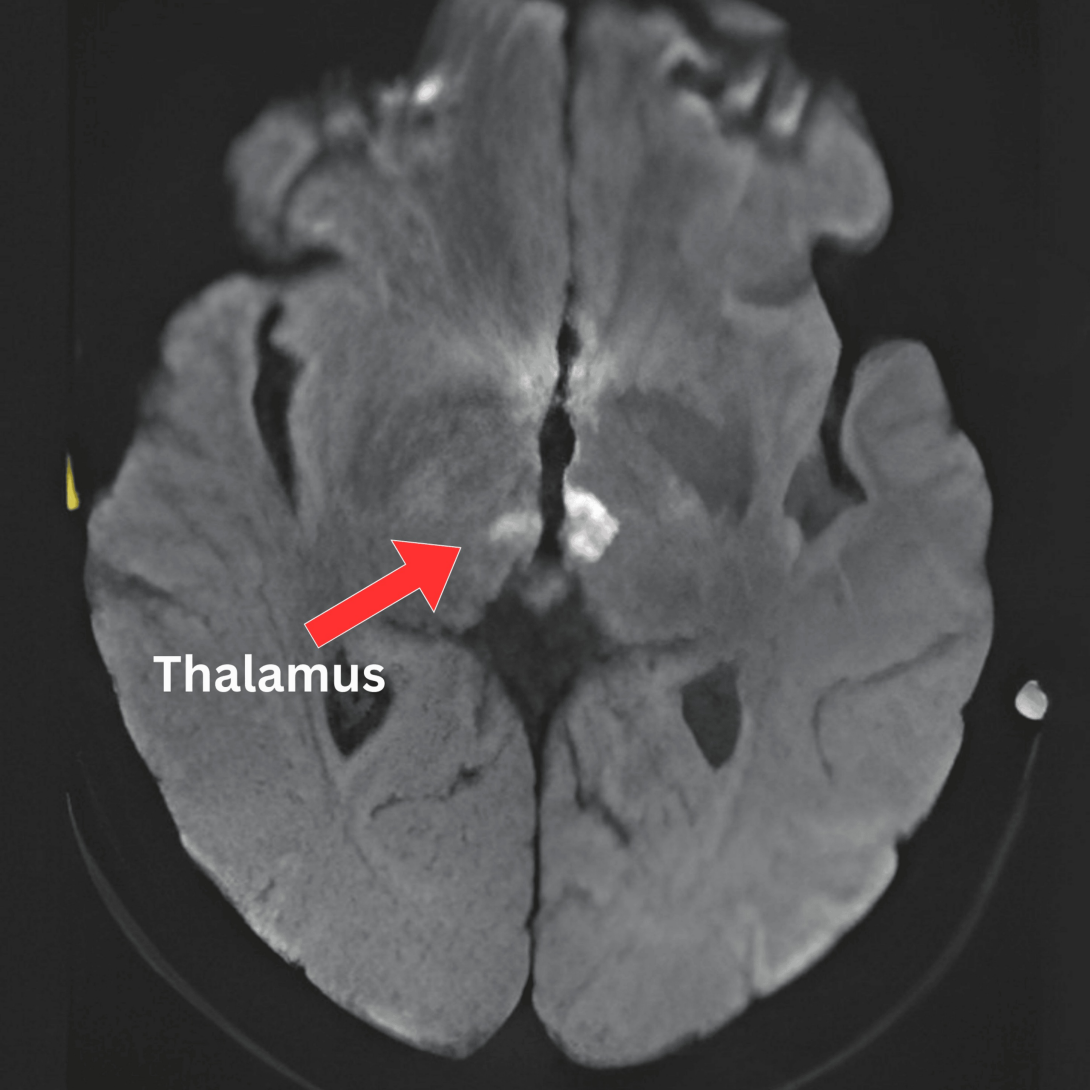
Non-contrast MRI brain DWI sequence showing corresponding diffusion restriction within bilateral thalami, consistent with cytotoxic oedema. DWI: diffusion-weighted imaging; MRI: magnetic resonance imaging.

The MRI of the brain demonstrated bilateral, symmetrical hyperintense signal abnormalities on T2-weighted and fluid-attenuated inversion recovery (FLAIR) sequences involving the medial thalami, periaqueductal grey matter, tectal plate, mammillary bodies, and pyramidal tracts (Figure 1). Diffusion-weighted imaging (DWI) showed corresponding diffusion restriction with low apparent diffusion coefficient (ADC) values, consistent with cytotoxic oedema (Figure 2). The symmetrical distribution across metabolically vulnerable regions and lack of a vascular territory pattern supported a metabolic encephalopathy rather than an acute infarction. No contrast enhancement or haemorrhage was identified. These findings, in conjunction with the clinical context and risk factors, were highly suggestive of acute WE.

High-dose parenteral thiamine therapy was initiated immediately following MRI, without delay for further investigations. The patient received intravenous Pabrinex high-potency, administered as two pairs three times daily (equivalent to thiamine 500 mg intravenously every eight hours) for five consecutive days, in accordance with NICE guidelines-based management. Thiamine was administered prior to any glucose-containing fluids.

Given documented hypomagnesaemia, oral magnesium aspartate 10 mmol sachets were administered twice daily concurrently for a total of five days, with regular monitoring of serum magnesium levels, recognising magnesium’s role as an essential cofactor for thiamine-dependent enzymatic activity.

After completing intravenous therapy, the patient was transitioned to oral thiamine 100 mg three times daily and Vitamin B Compound Strong one capsule once daily.

Hepatic encephalopathy was actively considered throughout admission. Normal ammonia levels, absence of asterixis, lack of response to supportive measures, and the characteristic MRI findings supported WE as the primary diagnosis. A therapeutic trial of lactulose was not pursued, given the low clinical suspicion and alternative unifying diagnosis.

**Figure 3:**
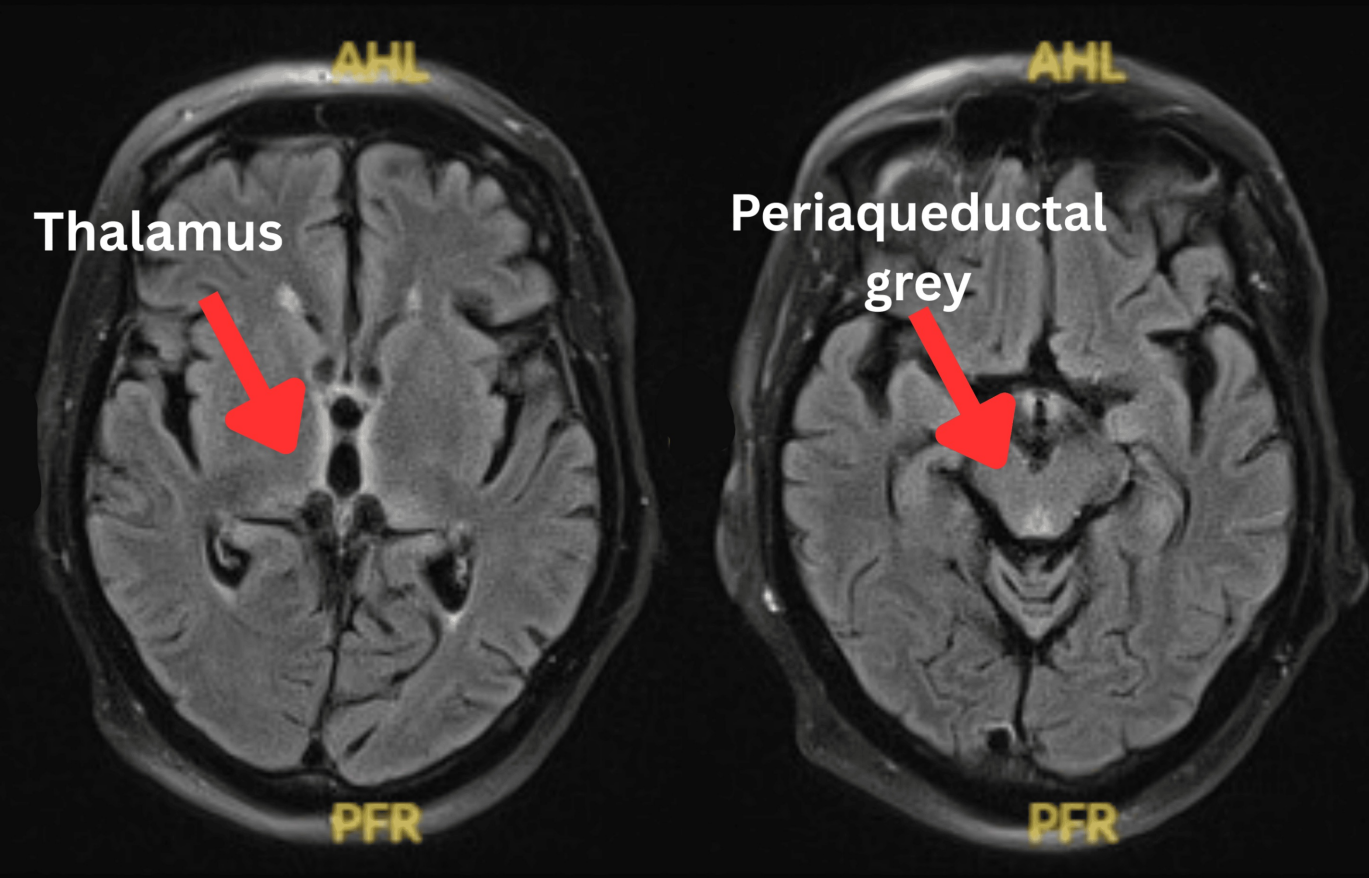
Follow-up MRI brain (axial FLAIR) five days after initiation of intravenous thiamine demonstrating marked regression of signal abnormalities. MRI: magnetic resonance imaging; FLAIR: fluid-attenuated inversion recovery; AHL: anterior horn of the left lateral ventricle; PFR: posterior frontal region.

A repeat MRI performed five days after initiation of thiamine therapy demonstrated marked regression of the previously observed signal abnormalities, confirming radiological reversibility (Figure 3).

Despite the recovery of consciousness and radiological improvement, the patient exhibited persistent neurological deficits. These included dysarthria, gait ataxia requiring assistance with mobilisation, and significant memory impairment with confabulation. Formal cognitive assessment using the Montreal Cognitive Assessment (MoCA) was performed following stabilisation and completion of intravenous thiamine therapy, yielding a score of 12/30, consistent with severe cognitive impairment.

Functional assessment demonstrated ongoing impairment in activities of daily living, necessitating multidisciplinary input. Given the persistence of cognitive and gait deficits beyond the acute phase, the findings were considered consistent with chronic cognitive sequelae of WE, rather than complete neurological recovery.

The patient was subsequently transferred to a specialist neurorehabilitation facility for ongoing cognitive and physical rehabilitation. Longer-term neuropsychological follow-up was planned, though extended longitudinal outcomes were not available at the time of reporting.

## Discussion

This case illustrates an extreme and clinically instructive presentation of WE masquerading as an acute cerebrovascular catastrophe. While WE is a recognised stroke mimic, most reported cases describe subacute neurological decline. In contrast, this patient experienced an abrupt and profound reduction in consciousness, closely simulating catastrophic stroke or brainstem pathology.

The patient did not exhibit the full classical triad of WE, lacking ophthalmoplegia throughout his presentation. This is consistent with existing literature demonstrating that isolated or incomplete presentations are common [2,3]. MRI was pivotal in establishing the diagnosis, demonstrating the characteristic symmetrical involvement of metabolically vulnerable brain regions. Diffusion restriction in WE reflects cytotoxic oedema due to metabolic failure rather than irreversible infarction, helping to distinguish WE from vascular stroke. The marked radiological improvement following thiamine administration is consistent with prior reports of imaging reversibility in WE [8,10].

Diffusion restriction in WE reflects reversible cytotoxic oedema rather than infarction and may resolve rapidly with thiamine replacement [11]. Importantly, this case highlights that radiological resolution does not reliably predict neurological or cognitive recovery. Despite dramatic imaging improvement, the patient demonstrated persistent cognitive impairment on formal assessment. These findings suggest that severe or abrupt presentations may be associated with lasting cerebral dysfunction even when treatment is initiated promptly [9,12].

Current guidelines advocate immediate parenteral thiamine administration when WE is suspected, without waiting for diagnostic confirmation, as delays can result in permanent disability or death [6,13]. Magnesium replacement is also essential, as deficiency impairs thiamine-dependent enzymatic activity [14].

This report reinforces the importance of early empirical treatment with high-dose parenteral thiamine and magnesium replacement when WE is suspected. However, it also underscores the need for cautious prognostication, as early radiological improvement may not translate into favourable long-term cognitive outcomes.

## Conclusions

WE should be considered in any patient presenting with acute unexplained neurological deterioration and risk factors for thiamine deficiency, even when classical features are incomplete. This case demonstrates that WE can present with abrupt coma and mimic catastrophic stroke, with characteristic MRI findings and rapid radiological reversibility following thiamine therapy. However, early imaging improvement does not reliably predict cognitive recovery. Clinicians should maintain a low threshold for immediate empirical thiamine and magnesium replacement and should exercise caution in interpreting radiological resolution as evidence of a favourable neurological outcome.
